# Bias reported by family caregivers in support received when assisting patients with cancer‐related decision‐making

**DOI:** 10.1002/cam4.5182

**Published:** 2022-08-29

**Authors:** J. Nicholas Dionne‐Odom, Katherine A. Ornstein, Andres Azuero, Erin R. Harrell, Shena Gazaway, Kristen Allen Watts, Deborah Ejem, Avery C. Bechthold, Kyungmi Lee, Frank Puga, Ellen Miller‐Sonet, Grant R. Williams, Erin E. Kent

**Affiliations:** ^1^ School of Nursing University of Alabama at Birmingham Birmingham Alabama USA; ^2^ Division of Gerontology Geriatrics, and Palliative Care School of Medicine University of Alabama at Birmingham Birmingham Alabama USA; ^3^ Center for Palliative and Supportive Care University of Alabama at Birmingham Birmingham Alabama USA; ^4^ Department of Geriatrics and Palliative Medicine Icahn School of Medicine at Mount Sinai New York New York USA; ^5^ Department of Psychology University of Alabama Tuscaloosa Alabama USA; ^6^ Division of Preventive Medicine School of Medicine University of Alabama at Birmingham Birmingham Alabama USA; ^7^ CancerCare New York New York USA; ^8^ Division of Hematology‐Oncology School of Medicine University of Alabama at Birmingham Birmingham Alabama USA; ^9^ Gillings School of Global Public Health University of North Carolina Chapel Hill Chapel Hill North Carolina USA

**Keywords:** bias, cancer, decision‐making, discrimination, family caregiver

## Abstract

**Background:**

Family caregivers play an increasing role in cancer treatment decision‐making. We examined bias reported by family caregivers in the support they and their patient received from their healthcare team when making these decisions, including associations with distress.

**Methods:**

Analysis of 2021 national survey data of family caregivers of patients with cancer (*N* = 2703). Bias experienced in decision support was assessed with the item: “Have you felt that the support you and the person with cancer have received for making cancer‐related decisions by your doctor or healthcare team has been negatively affected by any of the following?” Check‐all‐that‐apply response options included: age, race, language, education, political affiliation, body weight, insurance type or lack of insurance, income, religion, sexual orientation, and gender/sex. Chi‐square and regression analyses assessed associations between bias and caregiver distress (GAD‐2, PHQ‐2).

**Results:**

Of 2703 caregiver respondents, 47.4% (*n* = 1281) reported experiencing ≥1 bias(es) when receiving decision support for making cancer‐related decisions. Bias was more prevalent among younger caregivers, males, transwomen/men or gender non‐conforming caregivers, racial/ethnic minorities, and those providing care over a longer time period. The odds of having high anxiety (GAD‐2 scores ≥ 3) were 2.1 times higher for caregivers experiencing one type of bias (adjusted OR, 2.1; 95% CI, 1.6–2.8) and 4.2 times higher for caregivers experiencing ≥2 biases (adjusted OR, 4.2; 95% CI, 3.4–5.3) compared to none. Similar results were found for high depression scores (PHQ‐2 scores ≥ 3).

**Conclusions:**

Nearly half of caregivers involved in their care recipients' cancer‐related decisions report bias in decision support received from the healthcare team. Experiencing bias was strongly associated with high psychological distress.

## Graphical abstract

Using data from a nationally representative online panel survey of family caregivers of patients with cancer, we found that nearly half of caregivers involved in their care recipients’ cancer‐related decisions reported bias in the healthcare team decision support they and the patient received. Furthermore, experiencing this bias was associated with high caregiver psychological distress.

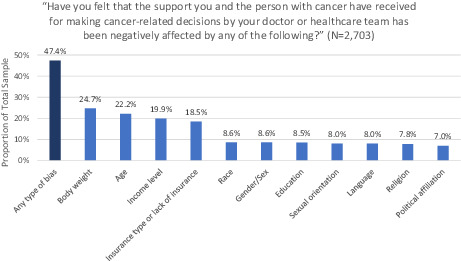

## 1 INTRODUCTION

A growing body of research reports that patients receiving healthcare services often experience negative differential treatment based on their group identity and other personal characteristics, such as age, race, gender/sex, and sexual orientation.^1^, ^2^, ^3^, ^4^ Understanding the extent of bias and discrimination in healthcare, including cancer care, has become an increasingly spotlighted area as it has well‐documented negative effects on an individual's psychological and physical health.^5^, ^6^, ^7^, ^8^ In addition to the negative impact this differential treatment has on psychological health, there is some emerging work showing the presence of discrimination when patients and families engage in shared decision‐making with clinicians about treatment.^9^ This is especially relevant in the context of cancer as family members and close friends (hereafter referred to as “family caregivers”) are often present and play a critical role in assisting patients with treatment and other health‐related decision‐making.^10^, ^11^, ^12^ In a large, multiregional study of 5284 patients with lung and colorectal cancer, nearly half (49.4%) reported sharing cancer treatment decisions equally with their family, and another 22.1% reported that they received some family input on these decisions.^10^ While the role of families in assisting patients with treatment and health‐related decision‐making is becoming clearer,^11^ less is known about the extent to which bias is perceived by families in the decision‐making context and what impact it may be having on their psychological health.

Hence, we used data from a large nationwide survey of family caregivers of individuals with cancer regarding their role in assisting patients with treatment and other health‐related decisions, including whether they believed the support they and the patient received from their healthcare team in making those decisions had been negatively impacted by their group characteristics (e.g., age, race, gender/sex). In addition to exploring the proportion and types of family caregivers who reported different types of bias, we hypothesized that perceiving bias would be associated with (a) high psychological distress and (b) higher distress if experiencing more than one type of bias.

## 2 METHODS

This was an analysis of de‐identified data from a national survey of family and friend caregivers of patients with cancer (*N* = 2703) conducted by CancerCare® and distributed for online completion between February and July, 2021. CancerCare is a national 501(c)3 nonprofit organization founded in 1944 that provides free, professional support services to patients with cancer and their families. The survey was developed in partnership with caregiving services and research experts (including JND‐O and EM‐S) to describe the role of cancer family caregivers in patient decision‐making and assess their needs for support. All survey items were reviewed by a CancerCare advisory board that included five professional patient advocates and piloted by a CancerCare social worker and other staff who provide counseling to cancer caregivers. Using a consumer insight and market research panel company (PureSpectrum), survey respondents were identified from consumer research panels who self‐identified as a family or friend caregiver of an individual with cancer who also reported involvement in making decisions regarding the person with cancer for whom they care. Because market research panels were used, it was not possible to calculate a traditional survey response rate. “Family caregiver” was defined in the survey as an individual providing unpaid support in the past 12 months to a family member or friend who is close to them, who has cancer, and who did not have to live in the same home. The survey sample had approximately 25% coverage in the U.S. Northeast, Midwest, Southeast, and Southwest/West. Participants were not paid for participation. The study was deemed exempt by the University of Alabama at Birmingham Institutional Review Board.

### 2.1 Variables

#### 2.1.1 Demographic and clinical characteristics

Sociodemographic (age, gender, race, ethnicity, education, geographic location, caregiver–patient relationship) and clinical information (cancer type and stage, length of time providing care) were self‐reported by caregiver respondents.

#### 2.1.2 Outcomes

Bias experienced in decision support was assessed with the item: “Have you felt that the support you and the person with cancer have received for making cancer‐related decisions by your doctor or healthcare team has been negatively affected by any of the following?” Check‐all‐that‐apply response options included: “age”, “race”, “language”, “education”, “political affiliation”, “body weight”, “insurance type or lack of insurance”, “income”, “religion”, “sexual orientation”, and “gender/sex”. A total of 11 areas of bias were possible and selected from a review of the literature by the study investigators.

Anxiety symptoms were measured using the 2‐item Generalized Anxiety Disorder (GAD‐2).^13^ Items ask about the frequency of experiencing feelings related to nervousness, anxiousness, and worrying over the past 2 weeks. Scores range from 0 to 6, with scores of 3 or higher representing a positive screen for possible generalized anxiety disorder (sensitivity = 86%; specificity = 83%).^14^ Depression symptoms were measured using the 2‐item Patient Health Questionnaire‐2 (PHQ‐2),^15^, ^16^ which has shown good sensitivity and specificity for detecting major depressive disorder (sensitivity = 72%; specificity = 85%) for scores ≥ 3.^17^ Items ask about the frequency over the past 2 weeks of experiencing little interest or pleasure in doing things and feeling down, depressed, or hopeless. Scores also range from 0 to 6, with scores of 3 or higher representing a positive screen for possible major depressive disorder.

## 3 STATISTICAL METHODS

Descriptive statistics were used to characterize demographic and clinical characteristics and proportions of respondents experiencing bias. Pearson chi‐square was performed to determine differences in experiencing any bias (compared to experiencing none) by caregiver sociodemographic characteristics. To help assess the magnitude of differences, Cramer's *V* was computed and interpreted using Cohen's guidelines (small ~ 0.1, medium ~ 0.3, large ~ 0.5).^18^ Independent samples t‐tests were used to examine mean differences in GAD‐2 and PHQ‐2 between respondents who reported a bias versus those who did not. The magnitude of the mean differences was assessed with Cohen's *d* (small ~ 0.2, medium ~ 0.5, large ~ 0.8). Binomial logistic regression was performed to ascertain the association between experiencing bias (experiencing no bias versus experiencing 1 area of bias only versus experiencing multiple [2 or more] areas of bias) and high anxiety (model 1) and depression (model 2) symptoms. High and low anxiety and depression scores were based on pre‐established clinical thresholds for the GAD‐2 and PHQ‐2 (scores ≥ 3 = high).^13^, ^15^ Adjusting caregiver covariates were selected based on their association with anxiety and depression symptoms in prior studies (age, race, ethnicity, education, income, geographic location, cancer stage, and length of time providing care).^19^ All analyses were conducted using IBM SPSS Statistics 25.

## 4 RESULTS

The sociodemographic and clinical characteristics of respondents' care recipients are shown in Table 1. Patients were mostly 45 or older (82%), over half female (51%), and mostly White (79%) from all areas of the United States including the Midwest (19%), Northeast (21%), South (38%), and West (22%). Patients had a wide range of solid‐tumor and hematologic cancers in both type and stage, with breast (21%), lung (14%), and prostate (13%) cancers representing the largest groups.

**TABLE 1:** Descriptive characteristics of patients as reported by caregiver respondents (*N* = 2703)

Characteristic	Frequency	%
Age		
18–24	61	2.3
25–34	148	5.5
35–44	262	9.7
45–54	475	17.6
55–64	664	24.6
65–74	634	23.5
75 and older	446	16.5
Gender		
Female	1390	51.4
Male	1248	46.2
Trans woman/man or gender non‐conforming	62	2.3
Race		
White	2128	78.7
African American/Black	311	11.5
Asian	133	4.9
Alaskan Native, American Indian, Native Hawaiian, or Pacific Islander	47	1.8
2 or more races	62	2.3
Region of the United States		
Midwest	515	19.3
Northeast	559	21.0
South	1005	37.8
West	583	21.9
Cancer type		
Bladder	85	3.2
Brain	98	3.6
Breast	567	21.0
Colon/rectal	184	6.8
Gynecologic	181	6.7
Head and neck	46	1.7
Kidney	81	3.0
Leukemia	264	9.8
Lung	375	13.9
Lymphoma	93	3.4
Melanoma	109	4.0
Multiple myeloma	51	1.9
Pancreatic	65	2.4
Prostate	343	12.7
Thyroid	68	2.5
Cancer stage—solid tumor		
In remission	96	3.6
1–2	970	35.9
3–4	1167	46.9
Cancer stage—hematologic		
In remission	51	1.9
0, 1, 2	178	6.6
3, 4	153	5.7

Caregivers (*N* = 2703) were a range of ages with individuals aged between 35 and 54 comprising the largest bracket (48%) (Table 2). Just over half were female (53%), less than half were male (45%) and small proportion were trans woman or trans man or gender non‐conforming (2%). Most were White (78%) and African American/Black (13%). About 16% of the sample was Hispanic/Latino. Caregivers came from a range of educational backgrounds with approximately 67% having a 4‐year college degree or higher. Most were employed full‐ or part‐time (82%), and a majority earned $75,000 or more in total household income (62%). Most lived in urban settings (83%). Caregivers identified most frequently as the child (33%), friend (25%), or spouse/partner (12%) of the person with cancer and most had been providing care between 1 and 3 years.

**TABLE 2:** Reported bias by caregiver sociodemographic characteristics

Characteristic	Total, *N* = 2703, %	Report ≥1 areas of bias^a^, *n* (%)	*p* value^b^	Cramer's *V* ^b^
Overall	2703 (100)	1281 (47.4)	—	—
Caregiver age				
18–34	812 (30.0)	467 (57.5)	<0.001	0.20
35–54	1307 (48.4)	640 (49.0)		
55 and older	578 (21.4)	170 (29.4)		
Caregiver gender				
Male	1224 (45.3)	650 (53.1)	<0.001	0.14
Female	1434 (53.1)	597 (41.6)		
Trans woman/man or gender non‐conforming	44 (1.6)	34 (77.3)		
Caregiver race				
White	2106 (77.9)	966 (45.9)	<0.01	0.08
African American/Black	342 (12.7)	187 (54.7)		
Asian	154 (5.7)	68 (44.2)		
Alaskan Native, American Indian, Native Hawaiian, or Pacific Islander	33 (1.2)	23 (69.7)		
Hispanic/Latino				
Yes	439 (16.2)	254 (57.9)	<0.001	0.09
No	2256 (83.5)	1022 (45.3)		
Caregiver education				
Post graduate degree	763 (28.2)	371 (48.6)	<0.01	0.08
Some post graduate	169 (6.3)	101 (59.8)		
College graduate (4 year)	896 (33.1)	424 (47.3)		
Vocational/Technical School (2 year)	158 (5.8)	61 (38.6)		
Some college	420 (15.5)	183 (43.6)		
High school graduate or less	293 (10.8)	140 (47.8)		
Caregiver employment				
Full or part‐time employment	2203 (81.5)	1096 (85.6)	<0.001	0.07
Retired	246 (9.1)	79 (6.2)		
Not employed	190 (7.0)	68 (5.3)		
Student	53 (2.0)	34 (2.7)		
Caregiver total household income				
<$75,000	997 (36.9)	461 (46.2)	0.27	0.02
≥$75,000	1672 (61.9)	810 (48.5)		
Location				
Urban	2253 (83.4)	1069 (47.4)	0.08	0.03
Rural or small town	351 (13.0)	149 (42.5)		
Caregiver‐patient relationship (The patient is the caregiver's…)				
Parent	892 (33.0)	410 (46.0)	0.02	0.07
Friend	676 (25.0)	335 (49.6)		
Spouse/partner	314 (11.6)	128 (40.8)		
Sibling	162 (6.0)	76 (46.9)		
Child	48 (1.8)	29 (60.4)		
Extended family (e.g., aunt/uncle, grandparent, cousin)	587 (21.7)	296 (50.4)		
Length of time providing care				
Up to 1 year	860 (31.8)	400 (46.5)	<0.01	0.07
1–3 years	1160 (42.9)	524 (45.2)		
3–5 years	339 (12.5)	165 (49.0)		
5 or more years	344 (12.7)	192 (55.8)		

^a^ Areas of bias: age, race, language, education, political affiliation, body weight, insurance type or lack of insurance, income level, religion, sexual orientation, gender/sex.

^b^ Comparing those experiencing none vs. ≥1 type of bias.

**FIGURE 1:**
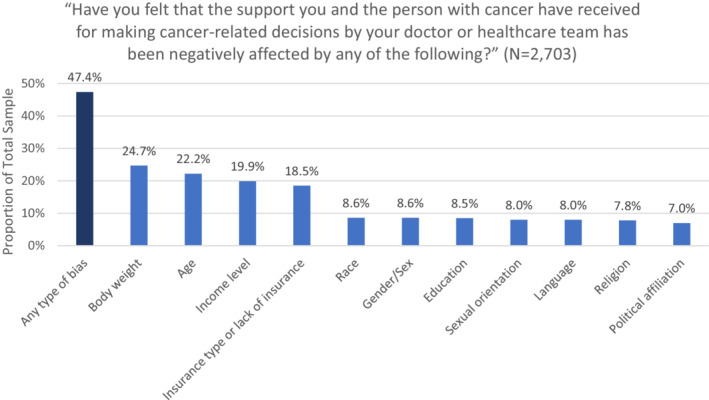
Self‐reported bias by cancer family caregivers (overall and by 11 bias areas)

Figure 1 summarizes self‐reported areas of bias by caregivers for the support received by the healthcare team for making cancer‐related decisions. Among all caregivers, 47% reported experiencing at least one type of bias. The most frequently reported biases were body weight (25%), age (22%), income level (20%), and insurance type/lack of insurance (19%). Proportions of reported bias for race, gender/sex, education, sexual orientation, language, religion, and political affiliation ranged from 7% to around 9%.

Table 2 shows differences in experiencing one or more areas of bias by caregiver sociodemographic characteristics. Larger proportions of caregivers who experienced bias were younger (58%), male (53%), trans woman/man or gender non‐conforming (77%), African American/Black (55%), Alaskan Native, American Indian, Native Hawaiian, or Pacific Islander (70%), Hispanic/Latino (58%), had some post‐graduate education (60%), were a parental family caregiver (60%), and had been providing care for ≥5 years (56%). Proportions of individuals by sociodemographic characteristic and individual bias area are included in the Supplementary Table.

Table 3 depicts unadjusted differences in GAD‐2 and PHQ‐2 mean scores by respondents who had and had not experienced different types of bias using independent samples *t*‐tests. Differences between groups were statistically significant for all types of bias (all *p*'s < 0.001), and effect size differences were medium to large for all comparisons (range: 0.44–0.73). Surpassing clinical cutoffs for high anxiety and depression symptoms were observed for most types of bias.

**TABLE 3:** Anxiety and depression scores by bias type^a^

Bias type	GAD Score, Mean (SD) (Range: 0–6 [low–high], ≥3 = clinically high)	*d* ^b^	PHQ‐2 Score, Mean (SD) (Range: 0–6 [low–high], ≥3 = clinically high)	*d* ^b^
Age				
Yes (*n* = 595)	2.9 (1.8)	0.48	2.7 (1.8)	0.48
No (*n* = 2068)	2.1 (1.9)		1.8 (1.8)	
Race				
Yes (*n* = 236)	3.4 (1.8)	0.66	3.0 (1.9)	0.62
No (*n* = 2427)	2.1 (1.8)		1.9 (1.8)	
Language				
Yes (*n* = 228)	3.3 (1.9)	0.62	3.2 (1.9)	0.71
No (*n* = 2435)	2.2 (1.9)		1.9 (1.8)	
Education				
Yes (*n* = 228)	3.2 (1.8)	0.59	3.0 (1.9)	0.60
No (*n* = 2435)	2.2 (1.9)		1.9 (1.8)	
Political affiliation				
Yes (*n* = 194)	3.4 (1.8)	0.64	3.2 (1.9)	0.71
No (*n* = 2469)	2.2 (1.9)		1.9 (1.8)	
Body weight				
Yes (*n* = 648)	2.9 (1.8)	0.44	2.7 (1.8)	0.48
No (*n* = 2015)	2.0 (1.9)		1.8 (1.8)	
Insurance type or lack of insurance				
Yes (*n* = 495)	3.1 (1.9)	0.60	2.8 (1.8)	0.53
No (*n* = 2168)	2.0 (1.8)		1.8 (1.8)	
Income level				
Yes (*n* = 526)	3.0 (1.8)	0.51	2.8 (1.9)	0.58
No (*n* = 2137)	2.0 (1.9)		1.8 (1.8)	
Religion				
Yes (*n* = 219)	3.4 (1.8)	0.65	3.2 (1.8)	0.69
No (*n* = 2444)	2.1 (1.9)		1.9 (1.8)	
Sexual orientation				
Yes (*n* = 217)	3.3 (1.8)	0.61	3.2 (1.8)	0.73
No (*n* = 2446)	2.2 (1.9)		1.9 (1.8)	
Gender/sex				
Yes (*n* = 237)	3.3 (1.9)	0.59	3.1 (1.8)	0.66
No (*n* = 2426)	2.1 (1.9)		1.9 (1.8)	

^a^ Independent samples *t*‐test.

^b^ Cohen's *d*; absolute values reported; *p*‐values for all comparisons <0.001.

Results from logistic regression models predicting high depression and anxiety symptoms are shown in Table 4. After adjusting for covariates, those experiencing one type of bias had 2.0 times higher odds of high anxiety symptoms compared to those reporting no bias; those with ≥2 areas of bias had 4.2 times higher odds of high anxiety symptoms compared to those reporting no bias. Similarly, those experiencing one area of bias had two times higher odds of high depression symptoms compared to those reporting no bias; those experiencing ≥2 areas of bias had 4.4 higher odds of high depression symptoms compared to those reporting no bias.

**TABLE 4:** Association between reported bias and clinically high GAD‐2 and PHQ‐2 scores

Variable	*B*	SE	OR	95% CI for OR		*p*‐value
High GAD‐2 scores (scores ≥ 3)^a^						
*Constant*	−0.62	0.22	0.54	n/a	n/a	0.004
Bias experienced in one area^b^	0.72	0.15	2.0	1.5	2.7	<0.001
Bias experienced in 2 or more areas^b^	1.4	0.11	4.2	3.4	5.2	<0.001
High PHQ‐2 scores (scores ≥ 3)^a^						
*Constant*	−0.88	0.22	0.42	n/a	n/a	<0.001
Bias experienced in one area^b^	0.69	0.15	2.0	1.5	2.7	<0.001
Bias experienced in 2 or more areas^b^	1.5	0.11	4.4	3.5	5.5	<0.001

^a^ Models adjusted for: caregiver age, gender, race, education, employment, income, time providing support, and patient's cancer stage.

^b^ Reference category: No bias experienced.

## 5 DISCUSSION

Using data from a national survey of over 2700 family caregivers of individuals with cancer, we evaluated the proportions and types of caregivers who reported bias in the healthcare team support they and their patient received when making cancer‐related decisions. Nearly half of cancer caregivers (47%) reported experiencing bias. The most frequently endorsed types of bias were body weight, age, income level, and insurance—biases which have all been observed to be highly prevalent in both the general population and by patients in the care received from healthcare clinicians.^20^, ^21^, ^22^, ^23^, ^24^ Furthermore, caregivers reporting bias were markedly more likely to have clinically high anxiety and depression symptoms. To our knowledge, these are the first data of this kind in the cancer caregiving decision‐making context and raise concerns about the pervasiveness of bias in decision support reported by families involved in patients' cancer‐related decisions. Such findings are especially concerning given how challenging cancer caregiving can be as evidenced by the high prevalence of depression and other outcomes.^19^, ^25^ Further investigation and a critical evaluation of clinician decision support practices is clearly warranted.

Caregivers who experienced high rates of bias in this study included groups for whom there is well‐documented historical and present‐day discrimination, including trans women, trans men, and gender non‐conforming individuals and individuals who are African American/Black, Alaskan Native, American Indian, Native Hawaiian, Pacific Islander, and Hispanic/Latino.^3^ Sociodemographic characteristics associated with high rates of bias in our sample that have been less frequently reported include younger individuals, men, those with some post‐graduate education, child family caregivers, and those providing care for longer periods of time. Of these, what was most unexpected was the finding that younger individuals and men reported higher rates of bias, when historically, older adults and women have faced higher rates of discrimination.^3^, ^20^ One possible explanation is that younger individuals and men may be culturally viewed as less conforming to the stereotypical image of caregiving, namely one that is often viewed as a traditionally middle‐to‐older age, female social role. Further work is needed to better understand these findings.

Our results showed a strong link between all areas of bias and heightened psychological distress, which is consistent with a large body of research to date. A meta‐analysis of 328 studies by Schmitt and colleagues found that perceived discrimination was significantly negatively correlated with measures of well‐being, such as self‐esteem, depression, anxiety, and life satisfaction (*r* = −0.23).^1^ Our results also demonstrated a more than four times heightened risk of high psychological distress for individuals experiencing multiple areas of bias. While individual biases have been the central focus of studies far more than the intersectionality of multiple bias types, research to date suggests that possessing multiple stigmatized identities and characteristics can have compounding negative effects on health.^26^, ^27^ It is important to note that the individual‐level traits that are subject to the biases explored in our study have varying outward degrees of visibility. For example, bias experienced toward one's gender/sex or race may have different contextual and behavioral features and outcomes compared to biases that are directed toward “invisible” or “concealable” traits such as one's political affiliation or income.^28^ Our findings strongly suggest that research and clinical approaches to promoting equity in cancer‐related decision‐making should incorporate an intersectional approach that considers the varying types of bias.^29^

There are several important limitations to this study that point toward key directions for future research. First, the data for this study are cross‐sectional, and hence, we are unable to draw conclusions about directionality between experiencing bias and psychological distress. It could be the case that individuals who are more distressed are more likely to anticipate and interpret discrimination in their interpersonal interactions.^30^, ^31^ Future work should address this by using longitudinal designs, which also characterize trajectories of distress over time. Second, our results do not tell us *how* the decision support by clinicians negatively affected the family and patient's actual decisions. A related question is how exactly did respondents note this negative impact in the actions and behaviors of the decision support received from the healthcare team? Different manifestations of discrimination have been described, including offensive or disparaging comments, negative assumptions about capability and intelligence, actions of exclusion or disregard, and lack of environmental accommodations.^32^ It is additionally important to highlight that while bias may not have been overtly intended by clinicians, bias can still be perceived and experienced by patients and families and exact deleterious effects on their care experiences and overall well‐being. Future work should ascertain which behaviors and features of decision support interactions with clinicians are being observed as indicative of bias, which can help guide targets for interventions. Third, the item eliciting experiences of bias may have been interpreted in different ways by respondents such that it confound clear interpretation of the meaning of responses. For example, the item wording makes it unclear if the bias was directed at the caregiver, their patient, or both. Also, given that “cancer‐related decision‐making” and “decision support” is a complex, psychological, and emotional process that spans across time, people, and settings, it is unclear how respondents were picturing their experience of bias based on this item; hence concern is warranted about the questionable face and content validity of this item. Given this, our findings might best be framed as exploratory and hypothesis‐generating for future work and not as firmly establishing prevalence rates of bias and bias types in cancer care. Future work on bias and discrimination in the healthcare decision‐making context should develop or employ more established instrumentation to assess these experiences. Fourth, we are unable to confirm how experiencing bias affects family and patient cancer‐related decision‐making, including the quality of the shared decision‐making process, patient–clinician trust, the choices that are offered and chosen, and how choices are implemented. While numerous frameworks have been developed to conceptualize discrimination in healthcare,^7^, ^33^, ^34^ a more granular understanding of the causal implications of bias on cancer‐related decision‐making processes and outcomes would provide a basis upon which interventions might be developed to lessen negative effects. Finally, the use of market research panels that favors individuals with internet access and precludes calculation of responses rates introduces a selection bias into this sample that, among other observed characteristics that differ from U.S. population indicators (e.g., income), limits the representativeness of the sample and generalizability of the findings.

In conclusion, our results from a large national survey of family caregivers of individuals with cancer who assist with cancer‐related decisions found that nearly half‐reported bias in the decision support they received from the healthcare team and this bias was highly associated with high psychological distress. Future research and clinical approaches to decision support need to consider holistic paradigms and system frameworks that specifically include an equity lens at clinician, system, and policy levels and aim at minimum to avoid harm due to discrimination in cancer‐related and shared decision‐making.

## AUTHOR CONTRIBUTIONS

**J Nicholas Dionne‐Odom:** Conceptualization (lead); formal analysis (lead); methodology (lead); writing – original draft (lead); writing – review and editing (equal). **Katherine Ornstein:** Conceptualization (supporting); formal analysis (supporting); methodology (supporting); writing – review and editing (supporting). **Andres Azuero:** Conceptualization (supporting); formal analysis (supporting); methodology (supporting); writing – review and editing (supporting). **Erin R Harrell:** Conceptualization (supporting); writing – review and editing (supporting). **Shena Gazaway:** Conceptualization (supporting); writing – review and editing (supporting). **Kristen Allen Watts:** Writing – review and editing (supporting). **Deborah B Ejem:** Writing – review and editing (supporting). **Avery C Bechthold:** Project administration (supporting); writing – review and editing (supporting). **Kyungmi Lee:** Writing – review and editing (supporting). **Frank Puga:** Conceptualization (supporting); writing – review and editing (supporting). **Ellen Miller‐Sonet:** Data curation (lead); funding acquisition (lead); project administration (lead); resources (lead); supervision (lead); writing – review and editing (supporting). **Grant R. Williams:** Writing – review and editing (supporting). **Erin Kent:** Conceptualization (supporting); formal analysis (supporting); methodology (supporting); visualization (supporting); writing – review and editing (supporting).

## FUNDING INFORMATION

The original CancerCare survey received funding support from Amgen, Merck, Regeneron, Eisai, Pfizer, Jazz, Astellas, and Glaxo‐Smith Kline. There was no research support for this analysis.

## CONFLICT OF INTEREST

All authors declare no conflict of interest.

## Supporting information


Appendix S1

## Data Availability

The data that support the findings of this study are available from CancerCare upon reasonable request.
