# Supplementary material for: Bias reported by family caregivers in support received when assisting patients with cancer‐related decision‐making
Source: Cancer Med. 2022 Aug 29;12(3):3567–76. doi: 10.1002/cam4.5182 (PMC9939189; doi:10.1002/cam4.5182)
Supplement: Supplementary file 1 — Appendix S1 [file CAM4-12-3567-s001.docx]

| **Supplementary Table.** Types of Self-Reported Bias by Caregiver Sociodemographic Characteristics | | | | | | | | | | | | |
| --- | --- | --- | --- | --- | --- | --- | --- | --- | --- | --- | --- | --- |
| **Characteristic** | **Total**  **N=2703, %** | **Age, %** | **Race, %** | **Language, %** | **Education, %** | **Political affiliation, %** | **Body weight, %** | **Insurance type or lack of insurance, %** | **Income level, %** | **Religion, %** | **Sexual orientation, %** | **Gender/**  **Sex, %** |
| Caregiver age |  |  |  |  |  |  |  |  |  |  |  |  |
| 18-34 | **812 (30.0)** | 24.6 | 13.5 | 11.0 | 12.8 | 9.7 | 30.3 | 25.2 | 24.5 | 10.5 | 11.8 | 12.3 |
| 35-54 | **1307 (48.4)** | 23.6 | 8.4 | 9.2 | 8.0 | 7.7 | 25.5 | 17.4 | 20.2 | 9.0 | 8.1 | 9.3 |
| 55 and older | **578 (21.4)** | 16.1 | 2.8 | 3.5 | 3.8 | 3.1 | 12.6 | 11.4 | 11.8 | 3.5 | 3.1 | 3.1 |
| Caregiver gender |  |  |  |  |  |  |  |  |  |  |  |  |
| Male | **1224 (45.3)** | 25.8 | 9.6 | 10.1 | 9.5 | 8.6 | 27.9 | 19.6 | 21.2 | 10.0 | 10.6 | 10.8 |
| Female | **1434 (53.1)** | 18.9 | 7.4 | 6.6 | 7.5 | 5.6 | 20.6 | 17.2 | 18.2 | 6.3 | 5.7 | 6.6 |
| Other (Trans woman/man or gender non-conforming) | **44 (1.6)** | 34.1 | 29.5 | 27.3 | 15.9 | 29.5 | 38.6 | 36.4 | 27.3 | 22.7 | 20.5 | 27.3 |
| Caregiver race |  |  |  |  |  |  |  |  |  |  |  |  |
| White | **2106 (77.9)** | 22.0 | 6.8 | 7.0 | 7.4 | 6.4 | 24.2 | 17.6 | 18.6 | 6.9 | 7.5 | 8.4 |
| African American/Black | **342 (12.7)** | 22.8 | 18.4 | 12.6 | 13.2 | 10.2 | 26.3 | 23.1 | 23.7 | 14.0 | 10.2 | 10.5 |
| Asian | **154 (5.7)** | 22.7 | 7.8 | 16.2 | 9.7 | 9.1 | 18.8 | 16.2 | 18.8 | 7.1 | 7.1 | 5.2 |
| Alaskan Native or American Indian | **24 (1.0)** | 33.3 | 29.2 | 16.7 | 25.0 | 12.5 | 25.0 | 33.3 | 37.5 | 16.7 | 16.7 | 16.7 |
| Native Hawaiian or Pacific Islander | **9 (<1.0)** | 22.2 | 33.3 | 33.3 | 22.2 | 33.3 | 44.4 | 44.4 | 44.4 | 22.2 | 44.4 | 44.4 |
| Hispanic/Latino |  |  |  |  |  |  |  |  |  |  |  |  |
| Yes | **439 (16.2)** | 24.1 | 13.0 | 15.0 | 13.4 | 12.1 | 25.7 | 23.0 | 23.0 | 12.3 | 12.5 | 13.0 |
| No | **2256 (83.5)** | 21.8 | 7.9 | 7.1 | 7.6 | 6.3 | 23.9 | 17.7 | 19.1 | 7.4 | 7.4 | 8.0 |
| Caregiver education |  |  |  |  |  |  |  |  |  |  |  |  |
| Post graduate degree | **763 (28.2)** | 22.8 | 8.9 | 9.0 | 8.1 | 7.9 | 27.3 | 16.5 | 17.3 | 8.5 | 9.3 | 10.6 |
| Some post graduate | **169 (6.3)** | 33.1 | 12.4 | 10.7 | 10.7 | 8.9 | 33.1 | 21.3 | 26.6 | 9.5 | 5.9 | 10.1 |
| College graduate (4 yr) | **896 (33.1)** | 21.5 | 8.5 | 7.3 | 8.4 | 5.9 | 24.2 | 19.5 | 19.5 | 7.7 | 8.1 | 7.6 |
| Vocational/Technical School (2 yr) | **158 (5.8)** | 16.5 | 9.5 | 9.5 | 7.0 | 7.6 | 16.5 | 15.8 | 18.4 | 8.9 | 7.6 | 9.5 |
| Some college | **420 (15.5)** | 21.9 | 7.1 | 8.1 | 9.5 | 6.4 | 19.0 | 19.0 | 18.8 | 8.1 | 6.2 | 6.4 |
| High school graduate | **258 (9.5)** | 18.6 | 8.1 | 8.1 | 7.8 | 9.7 | 20.9 | 20.5 | 24.0 | 7.8 | 7.8 | 8.9 |
| Some high school or less | **35 (1.3)** | 34.3 | 17.1 | 22.9 | 14.3 | 17.1 | 37.1 | 20.0 | 22.9 | 14.3 | 25.7 | 22.9 |
| Caregiver total household income |  |  |  |  |  |  |  |  |  |  |  |  |
| <$75,000 | **997 (36.9)** | 20.5 | 8.6 | 8.6 | 8.8 | 7.6 | 21.1 | 20.4 | 21.9 | 8.9 | 7.9 | 8.7 |
| ≥$75,000 | **1672 (61.9)** | 23.6 | 9.0 | 8.6 | 8.4 | 7.2 | 26.3 | 17.6 | 18.5 | 8.3 | 8.4 | 8.9 |
| Location |  |  |  |  |  |  |  |  |  |  |  |  |
| Urban | **2253 (83.4)** | 22.1 | 8.9 | 8.9 | 8.8 | 7.6 | 24.2 | 19.1 | 19.8 | 8.6 | 8.9 | 9.1 |
| Rural or small town | **351 (13.0)** | 21.1 | 6.6 | 5.1 | 4.6 | 4.6 | 19.9 | 15.1 | 18.8 | 4.6 | 4.3 | 6.0 |
| Caregiver-patient relationship (The patient is the caregiver’s…) |  |  |  |  |  |  |  |  |  |  |  |  |
| Parent | **892 (33.0)** | 25.8 | 8.4 | 7.6 | 7.3 | 5.5 | 21.2 | 17.6 | 16.7 | 6.7 | 6.2 | 6.6 |
| Friend | **676 (25.0)** | 18.3 | 9.0 | 8.6 | 10.1 | 8.7 | 28.3 | 19.4 | 21.6 | 10.4 | 11.2 | 9.9 |
| Spouse/partner | **312 (11.5)** | 20.5 | 8.0 | 8.3 | 5.8 | 8.3 | 17.6 | 17.0 | 17.6 | 10.3 | 9.0 | 9.0 |
| Sibling | **162 (6.0)** | 15.4 | 6.8 | 8.0 | 9.3 | 6.8 | 24.7 | 19.1 | 24.1 | 6.8 | 9.9 | 11.7 |
| Child | **48 (1.8)** | 31.8 | 16.7 | 18.8 | 22.9 | 14.6 | 33.3 | 33.3 | 27.1 | 16.7 | 16.7 | 22.9 |
| Extended family (e.g., aunt/uncle, grandparent, cousin) | **553 (20.5)** | 25.0 | 9.6 | 9.4 | 9.2 | 7.6 | 28.0 | 18.8 | 21.9 | 7.2 | 6.3 | 9.2 |
| Length of time providing care |  |  |  |  |  |  |  |  |  |  |  |  |
| Up to 1 year | **860 (31.8)** | 21.2 | 7.1 | 6.4 | 8.7 | 7.1 | 23.7 | 18.8 | 21.6 | 7.6 | 7.4 | 8.6 |
| 1 to 3 years | **1160 (42.9)** | 20.7 | 7.7 | 7.3 | 8.1 | 6.0 | 24.4 | 17.2 | 17.8 | 7.1 | 7.0 | 7.7 |
| 3 to 5 years | **339 (12.5)** | 26.8 | 10.3 | 10.6 | 7.7 | 8.0 | 21.2 | 20.1 | 17.4 | 6.8 | 7.7 | 8.3 |
| 5 or more years | **344 (12.7)** | 25.9 | 15.1 | 15.7 | 10.5 | 11.6 | 27.9 | 20.9 | 23.8 | 15.4 | 14.5 | 14.0 |
| Patient proximity (traveling by car) |  |  |  |  |  |  |  |  |  |  |  |  |
| Lives with caregiver in same home | **979 (36.2)** | 22.1 | 10.0 | 10.3 | 9.4 | 9.4 | 22.7 | 20.8 | 20.2 | 9.6 | 9.3 | 9.0 |
| Less than 15 minutes | **904 (33.4)** | 22.8 | 6.5 | 5.4 | 6.6 | 5.5 | 24.8 | 14.6 | 17.5 | 5.6 | 7.3 | 8.1 |
| Between 15 and 1 hour | **736 (27.3)** | 22.1 | 9.8 | 9.6 | 10.3 | 7.2 | 27.3 | 20.4 | 22.1 | 9.5 | 8.6 | 9.9 |
| More than 1 hour away | **72 (2.7)** | 20.8 | 8.3 | 11.1 | 2.8 | 4.2 | 9.7 | 20.8 | 18.1 | 6.9 | 1.4 | 5.6 |
